# Highly heterogeneous epitaxy of flexoelectric BaTiO_3-δ_ membrane on Ge

**DOI:** 10.1038/s41467-022-30724-7

**Published:** 2022-05-30

**Authors:** Liyan Dai, Jinyan Zhao, Jingrui Li, Bohan Chen, Shijie Zhai, Zhongying Xue, Zengfeng Di, Boyuan Feng, Yanxiao Sun, Yunyun Luo, Ming Ma, Jie Zhang, Sunan Ding, Libo Zhao, Zhuangde Jiang, Wenbo Luo, Yi Quan, Jutta Schwarzkopf, Thomas Schroeder, Zuo-Guang Ye, Ya-Hong Xie, Wei Ren, Gang Niu

**Affiliations:** 1 Electronic Materials Research Laboratory, Key Laboratory of the Ministry of Education & International Center for Dielectric Research, School of Electronic Science and Engineering & The International Joint Laboratory for Micro/Nano Manufacturing and Measurement Technology, Xi’an Jiaotong University, Xi’an, 710049 China; 2 State Key Laboratory of Functional Materials for Informatics, Shanghai Institute of Microsystem and Information Technology, Chinese Academy of Science, Shanghai, 200050 China; 3 Suzhou Institute of Nano-Tech and Nano-Bionics, Chinese Academy of Sciences, Suzhou, 215123 China; 4 The State Key Laboratory for Manufacturing Systems Engineering & The International Joint Laboratory for Micro/Nano Manufacturing and Measurement Technology, Xi’an Jiaotong University, Xi’an, 710049 China; 5 State Key Laboratory of Electronic Thin Films and Integrated Devices, University of Electronic Science and Technology of China, Chengdu, 611731 China; 6 School of Microelectronics, Xidian University, Xi’an, 710071 China; 7 Leibniz-Institut für Kristallzüchtung, Max-Born-Straße 2, Berlin, 12489 Germany; 8 Department of Chemistry and 4D LABS, Simon Fraser University, Burnaby, BC V5A 1S6 Canada; 9 Department of Materials Science and Engineering, University of California, Los Angeles, CA 90095 USA

**Keywords:** Nanoscale materials, Theory and computation, Graphene

## Abstract

The integration of complex oxides with a wide spectrum of functionalities on Si, Ge and flexible substrates is highly demanded for functional devices in information technology. We demonstrate the remote epitaxy of BaTiO_3_ (BTO) on Ge using a graphene intermediate layer, which forms a prototype of highly heterogeneous epitaxial systems. The Ge surface orientation dictates the outcome of remote epitaxy. Single crystalline epitaxial BTO_3-δ_ films were grown on graphene/Ge (011), whereas graphene/Ge (001) led to textured films. The graphene plays an important role in surface passivation. The remote epitaxial deposition of BTO_3-δ_ follows the Volmer-Weber growth mode, with the strain being partially relaxed at the very beginning of the growth. Such BTO_3-δ_ films can be easily exfoliated and transferred to arbitrary substrates like Si and flexible polyimide. The transferred BTO_3-δ_ films possess enhanced flexoelectric properties with a gauge factor of as high as 1127. These results not only expand the understanding of heteroepitaxy, but also open a pathway for the applications of devices based on complex oxides.

## Abstract (web summary)

The integration of epitaxial complex oxides on semiconductor and flexible substrates is required but challenging. Here, the authors report the highly heterogeneous epitaxy of transferrable BaTiO_3-δ_ membrane with enhanced flexoelectricity on Ge (011).

## Introduction

Future information society featuring “Internet of Things” and artificial intelligence requires the integration of multiple functionalities with semiconductor substrates like silicon (Si), germanium (Ge) and gallium arsenide (GaAs), such as sensing, micro-electromechanical systems, data storage, etc. Meanwhile, emerging wearable devices urgently demand the fabrication of functional layers on flexible substrates. Perovskite functional oxides with the ABO_3_ structure, represented by barium titanate (BaTiO_3_, BTO), have therefore attracted significant attention because they exhibit a wide spectrum of functionalities like ferroelectricity, piezoelectricity, non-linear optics, and flexoelectricity^1–4^.

Considering that the functionalities of perovskite oxides generally stem from the lattice asymmetry or the charge movement, the epitaxial single crystalline form shows optimal functionalities^5,6^. However, the heteroepitaxy of single-crystalline oxides on Si, Ge, or other semiconductor substrates is highly challenging, because semiconductor surfaces are easily oxidized and the subsequently formed amorphous interfacial layers can lead to the formation of polycrystalline or textured oxides^7,8^. A widely accepted solution is the utilization of a combination of alkaline earth metals (such as Ba and Sr) as sub-monolayer passivation layers, and the strict control of the interface and oxide growth conditions (such as temperature and oxygen partial pressure). For this purpose, complex ultra-high vacuum equipment like molecular beam epitaxy (MBE) are commonly required^9^. Moreover, it is even more difficult to grow epitaxial oxide films on flexible substrates which are mainly organic polymers with melting temperatures lower than 300 °C. The growth temperatures for well-crystallized oxides are typically higher than 500 °C^10^.

Recent years have witnessed the development of a number of solutions to overcome the above-mentioned difficulties, e.g., by the fabrication of single-crystalline oxide membranes and then the transfer of them onto required targeting substrates including semiconductor and flexible ones. For example, epitaxial BTO thin films were grown on sacrificial layers, which were subsequently dissolved in solvents (e.g., the mixture of KI and HCL for La_0.67_Sr_0.33_MnO_3_^11,12^, water for Sr_3_Al_2_O_6_^13–15^). Then released BTO membranes could be transferred to foreign substrates. Nevertheless, such methods have encountered difficulties in achieving large-area films and in maintaining the high quality of oxide films during the sacrificial layer etching procedure in a corrosive solution.

Remote epitaxy is an alternative method for the fabrication of epitaxial oxides. It uses graphene as an intermediate layer to grow epi-layers on iso-type substrates^16^. For example, remote epitaxy of semiconductor/semiconductor systems of GaN/SiC, GaAs/GaAs^16,17^, and of oxide/oxide systems like BTO/STO, STO/STO, and CoFeO_3_/STO^18–20^ have been reported. It was found that the periodic surface potential of the substrate still has the influence on adatoms on the top surface of one or two monolayers of graphene, guiding them to form crystalline structures. It has been shown that the polarity of the substrate is a dominating factor in the outcome of remote epitaxy^21^. Furthermore, the weak van der Waals force between graphene and substrates or epi-layers enables the peeling off and transfer of epitaxial oxide layers onto arbitrary substrates.

From an epitaxy point of view, Ge serves as a good substrate for the growth of high-quality BTO because of the quasi-lattice-match characteristic (with a lattice mismatch of only 0.08%^22–24^. Recent success in the growth of wafer-scale high-quality graphene on Ge (001) and Ge (011) substrates, therefore, makes Ge a great choice for the remote epitaxy of BTO^25,26^.

However, BTO and Ge possess highly heterogeneous characteristics in terms of lattice and chemistry, therefore the remote epitaxy of BTO on Ge has rarely been reported and the understanding of such oxide/graphene/semiconductor systems is poor. Many issues remain to be resolved, in particular, (i) whether graphene with good impermeability^27^ can play a passivation role in oxide/semiconductor heteroepitaxy; (ii) the influence of Ge surface orientations, which have different electrostatic potentials, on remote epitaxy needs to be studied; (iii) the nucleation and strain relaxation of oxides on graphene during remote epitaxy is unknown; and (iv) the functional properties especially the flexoelectricity of remote epitaxial BTO thin films need to be further explored. It is worth noting here that oxygen-deficient BTO, i.e., BTO_3-δ_ (which probably undergoes the phase transition from a tetragonal one to a cubic one), has been reported to show good flexoelectricity and the oxygen vacancies (${V}_{O}^{\bullet \bullet }$) strongly impact the flexoelectric properties due to the altering of the polarization and strain gradient of BTO^28^.

In this work, we achieved the remote epitaxy of BTO_3-δ_ films on the Ge (011) substrate covered by a directly chemical vapor deposition (CVD)-grown single-layer graphene. Such BTO_3-δ_ films were successfully exfoliated and transferred to the Si substrate and the polyimide (PI) flexible substrate and show good flexoelectric properties. The impact of the Ge surface orientation on the remote epitaxy of BTO was clarified based on both theoretical calculations and experiments. Graphene plays a crucial role in the passivation of the Ge surface (from oxidizing). The initial nucleation and the lattice relaxation as a function of the film thickness, as well as the crystallographic properties of remote epitaxial BTO_3-δ_ films were studied in detail. The strict control of the oxygen partial pressure assures the sharp interface and results in the good flexoelectricity of BTO_3-δ_ films. Our results not only solve fundamental issues of the remote epitaxy of highly heterogeneous BTO/Ge systems, such as the impacts of the surface orientation and the strain relaxation, but also open a pathway to the fabrication of oxide membranes for the monolithic integration on Si and flexible devices.

## Results and discussion

### Impact of Ge surface orientation on remote epitaxy

**Fig. 1:**
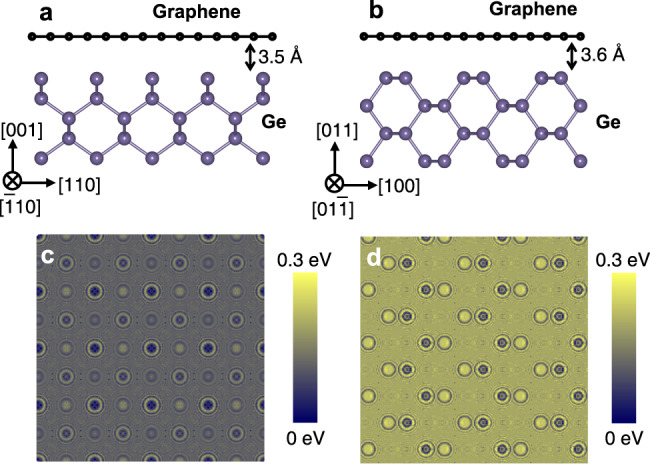
DFT calculations of surface potential fluctuations of Ge (001) and Ge (011). Sketches of graphene/Ge systems for **a** Ge (001) and **b** Ge (011). DFT-calculated electrostatic potential maps at *z* = 0.7 nm away from the Ge (001) (**c**) and Ge (011) (**d**) surfaces. The contrast of the images shows the potential fluctuations with the energy from low (gray) to high (yellow).

BTO films were grown on single-layer graphene-covered Ge substrates with surface orientations of (001) and (011). The graphene monolayers were directly grown on the Ge (001) and (011) substrates^25,26,29^ rather than being transferred. They showed flat morphology with few defects (e.g., pinholes, cracking, and wrinkles), as revealed by atomic force microscopy (AFM) and Raman spectroscopy characterizations (Fig. S1 in Supplementary Information (SI)). In order to evaluate the potential capability of Ge substrates with different orientations for the remote epitaxy of BTO films, ab initio density functional theory (DFT) calculations were performed for the spatial potential fluctuations, as shown in Fig. 1. Figure 1a, b indicates the atomic models of graphene/Ge (001) and graphene/Ge (011) systems, respectively. The electrostatic potential fluctuation distributions through the monolayer graphene at the plane 0.7 nm away from Ge substrate surface^30,31^ were shown in Fig. 1c, d for Ge (001) and Ge (011), respectively (more details can be found in supporting information). It can be seen that the potential map of Ge (001) shows large-area low energy (gray), whereas that of Ge (011) reveals a much higher energy (yellow). This indicates that the Ge surface orientation strongly impacts the surface electronic structure and thus influences the range of the chemical bonds. This difference between Ge (001) and Ge (011) would probably result in different behavior of the subsequently grown BTO. In order to understand the variation of the electrostatic potential distribution as a function of the distance from the Ge substrate, we further calculated the potential fluctuation maps at the plane 1.0 nm away from the Ge substrate, i.e., for adatoms on the top surface of two layers of graphene on Ge substrate^32^ (more details can be found in SI). The results were shown in Fig. S3 which reveals quite weak potential fluctuation maps for both Ge (001) and Ge (011).

Prior to the BTO deposition, an in situ pretreatment was performed at a high temperature of 650 °C in the PLD high vacuum chamber (more details see Method section) in order to remove possible surface contaminations and to examine the graphene quality at high temperatures. The surface morphology of the Ge (001) and Ge (011) substrate was then examined using AFM, as shown in Fig. S5a, b, respectively, which clearly exhibit flat surfaces with decreased surface root mean square (RMS) roughness compared to as-received substrates and the graphene monolayers remain intact with some wrinkles. Raman spectra were also recorded on the pre-treated samples and the results were shown in Fig. S5c, d, which indicates that the graphene monolayer remains intact without oxidization. It is noted here that even the introduction of a brief exposure to O_2_ at 650 °C would induce strong oxidation of graphene/Ge surface leading to intense density of holes in the graphene due to the volatile feature of GeO_*x*_, as shown in Fig. S6.

**Fig. 2:**
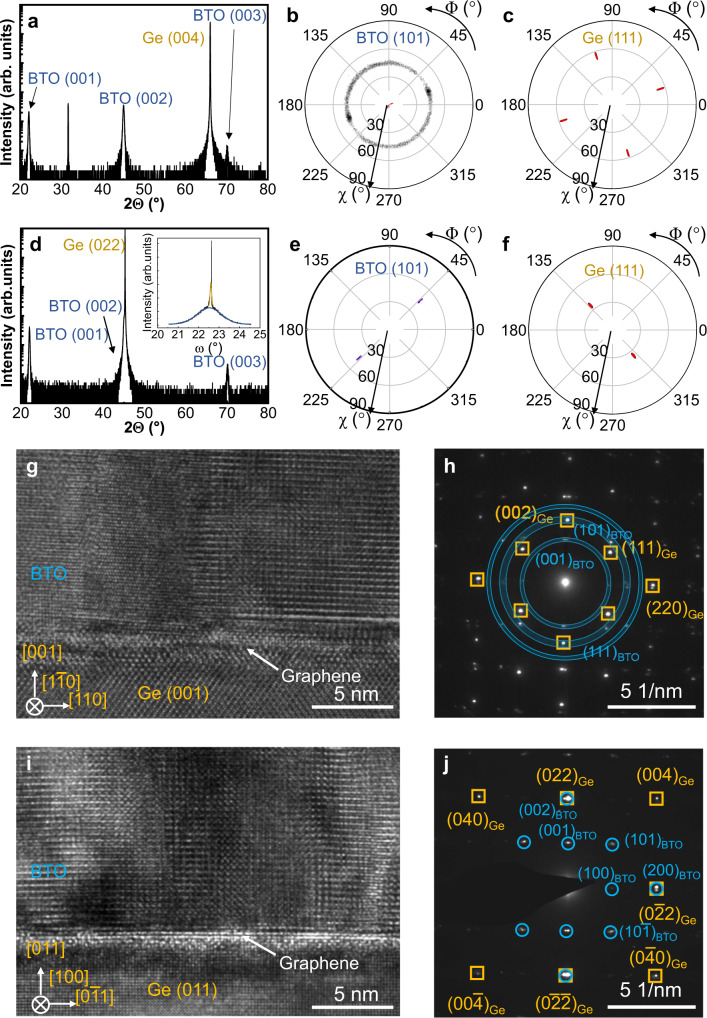
Crystallographic properties of BTO films on graphene/Ge. XRD results of BTO/graphene/Ge (001) heterostructure: **a** specular 2*θ* scan and pole figures using **b** BTO (101) Bragg conditions and **c** Ge (111) Bragg conditions. XRD results of BTO/graphene/Ge (011) heterostructure: **d** specular 2*θ* scan and pole figures using **e** BTO (101) Bragg conditions and **f** Ge (111) Bragg conditions. **g** Cross-sectional TEM image of the BTO /graphene/Ge (001) heterostructure. **h** SAED pattern of the sample is shown in **g**. Orange squares mark Ge diffraction dots while blue rings denote the BTO diffraction dots region. **i** Cross-sectional TEM image of the BTO/graphene/Ge (011) heterostructure. **j** SAED pattern of the sample shown in **i**. Orange squares mark Ge diffraction dots while blue circles denote BTO diffraction dots.

The crystallographic properties of the 90 nm-thick BTO films grown on graphene/Ge (001) and graphene/Ge (011) substrates after pre-treatment were examined by X-ray diffraction (XRD) and transmission electron microscopy (TEM) and the results were shown in Fig. 2. Figure 2a shows the specular out-of-plane (OP) XRD 2*θ* scan of BTO/graphene/Ge (001) using Ge (004) Bragg reflection conditions. It can be seen that only BTO (00l) diffraction peaks appear along with the Ge (004) reflection at 2*θ* = 66.01°. The crystallinity of such (001)-oriented BTO film was further examined by XRD pole figure (PF) measurements. Figure 2b shows the PF result using BTO (101) Bragg diffraction conditions, which indicates a ring structure at *χ* = 45°. Figure 2c shows the PF result of the Ge (001) substrate using Ge (111) Bragg diffraction conditions, which reveals the cubic structure of Ge lattice with four-fold spots at *χ* = 35.27°. This indicates that the (001) oriented BTO film is textured on graphene/Ge (001). Similarly, Fig. 2d–f shows XRD results of the BTO/graphene/Ge (011) sample. The specular XRD 2*θ* scan shown in Fig. 2d suggests that BTO is also (001) oriented on graphene/Ge (011). Furthermore, the PF results using BTO (101) and Ge (111) Bragg diffraction conditions shown in Fig. 2e, f, respectively, indicate that BTO was epitaxially grown with an epitaxial relationship of [100] BTO (001) || [110] Ge (011). More XRD investigations including *φ* scan and in-plane (IP) measurements were also performed and the results were shown in Figs. S7, S8, and S9, respectively, which further confirmed the above observation. The rocking curve of the remote epitaxial BTO_3-δ_ film is shown in Fig. 2d inset. There are two components corresponding to the Ge (022) peak (sharper) and BTO (002) peak (wider), respectively. The full width at half maximum of the BTO (002) peak is only 1.2°. It is worth noting here that the direct growth of BTO on bare Ge (011) substrate without the graphene monolayer leads to polycrystalline BTO films due to the oxidation of the Ge surface at the very beginning of the growth, which indicates the passivation role of the graphene layer. More details can be found in Fig. S10 in SI. Moreover, interestingly, it was reported that BTO films grown on bare Ge (001) substrates using interface engineering were single crystalline^33^ whereas BTO films grown on the graphene/SiO_2_/Si template were similarly textured with (001)-orientation, which reveals that graphene layer hinders the epitaxy of BTO on Ge (001) but induces the textured structure of BTO^34^.

The crystallographic properties of BTO films on graphene/Ge were further examined using high-resolution TEM (HRTEM) and the results were shown in Fig. 2g–j. Figure 2g exhibits the HRTEM image of the BTO/graphene/Ge (001) heterostructure, which shows different grains in the BTO film and a sharp interface without any oxidized layer. The selected area electron diffraction (SAED) pattern of the same sample was shown in Fig. 2h, which reveals two series of BTO diffraction spots. These observations corroborate that BTO on graphene/Ge (001) is textured and the graphene layer indeed plays a surface passivation role. On the contrary, the BTO film grown on graphene/Ge (011) shows an epitaxial characteristic without grains, as revealed by HRTEM shown in Fig. 2i. TEM energy dispersive X-ray spectroscopy (EDX) measurement (see Fig. S11 in SI) was also performed on BTO/graphene/Ge (011) and it shows a sharp interface without an amorphous layer nor interdiffusion of BTO to Ge, or vice versa. In particular, the carbon (C) element signal from the graphene single layer can be clearly identified at the BTO/Ge interface (Fig. S11). The SAED image shown in Fig. 2j indicates only one series of BTO diffraction spots, further confirming the remote epitaxy of the BTO on graphene/Ge (011). It is noted here that SAED patterns obtained from several randomly selected locations across the sample surface show identical characteristics, which stands as an evidence of the large-scale single crystalline films. Considering the theoretical calculation results, it is probable that the greater potential fluctuation of Ge (011) surface compared to that of Ge (001) surface leads to the remote epitaxy of BTO.

### Graphene passivation

In order to further investigate the surface passivation role of graphene monolayer, the 90 nm-thick BTO films directly grown on Ge (011) substrates using exactly the same growth conditions were studied by XRD and HRTEM and the results were shown in Fig. S10a, b, respectively. Both characterizations reveal that the BTO film directly grown on Ge (011) is polycrystalline and there exists an amorphous interfacial layer. This evidently indicates the important passivation role of graphene, which protects Ge (011) surface from oxidizing at the beginning of BTO growth at a relatively high temperature. It is interesting to point out that the BTO directly grown on Ge (001) substrate, however, shows an epitaxial feature^33^. This can be possibly attributed to a more active feature of Ge (011) compared to Ge (001), and further confirms the passivation role of graphene. It is also noted here that additional introduction of oxygen during the BTO growth will result in Ge oxidization despite the graphene passivation. Therefore, an interface engineering was carried out by limiting the oxygen partial pressure, which led to ${V}_{O}^{\bullet \bullet }$ in the BTO films. According to the XPS measurements shown in Fig. S12, the titanium (Ti) element shows both 3+ and 4+ valence states, indicating that the obtained films were BTO_3-δ_ with ${V}_{O}^{\bullet \bullet }$.

### Growth mode and relaxation

**Fig. 3:**
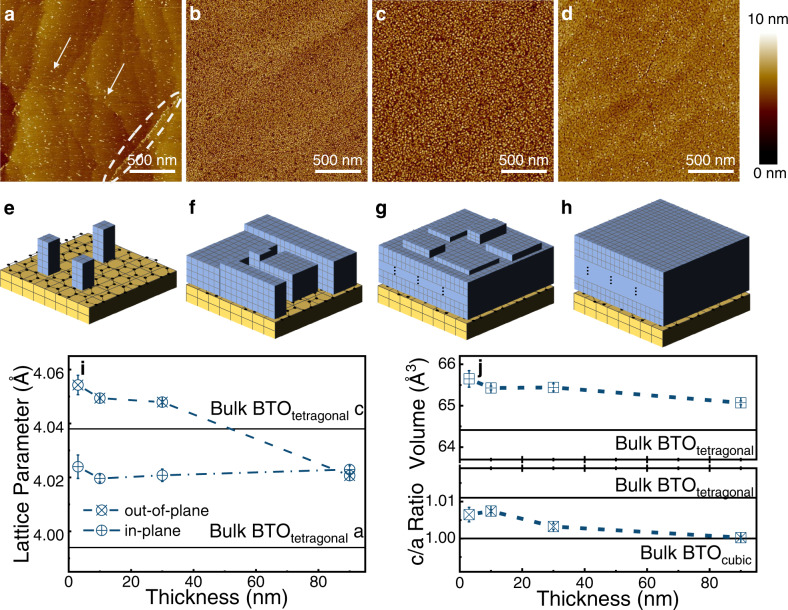
Growth mode and strain relaxation of remote epitaxial BTO films. AFM images of BTO films grown with different equivalent thicknesses: **a** 0.3 nm; arrows mark two of the separated islands and the wrinkle serving as the nucleation center was denoted; **b** 3 nm, **c** 10 nm, and **d** 90 nm. The schematic illustration of the film growth process: **e** Three-dimensional epitaxial islands nucleation following the Volmer-Weber mode, corresponding to **a**; **f** islands coalescing, corresponding to **b**; **g** and **h** formation of a flat film, corresponding to **c** and **d**. **i** Evolution of the lattice parameter as a function of the film thickness. The error bars are defined by the peak fitting of the XRD results shown in Fig. S15. **j** Evolution of the c/a ratio (lower) and lattice volume (upper) as a function of the film thickness. The error bars were calculated based on the results in **i**.

From the heteroepitaxy point of view, it is of great interest to understand the growth mode and the strain relaxation of remote epitaxial BTO_3-δ_ films on Ge (011). Therefore, four BTO_3-δ_ films with different equivalent thicknesses of 0.3 nm, 3 nm, 10 nm, 30 nm, and 90 nm were grown on graphene/Ge (011) and investigated in detail. Figure 3a–d shows the film morphology of 0.3 nm, 3 nm, 10 nm, and 90 nm thick BTO_3-δ_ samples obtained by AFM measurements, respectively. It can be seen in Fig. 3a that BTO_3-δ_ islands (two of them were marked by arrows) were randomly distributed on the graphene/Ge (011) surface, and aggregated along a graphene wrinkle at the right-bottom part (marked by a dotted oval). In addition, the atomic-scale structure of the Ge (011) surface can still be observed (see Fig. S1b for the initial graphene/Ge (011) surface for comparison). A line profile of the surface morphology (see Fig. S14) was performed and reveals that the average height of BTO_3-δ_ islands is approximately 2.5 nm. Such an average height is much larger than the expected equivalent thickness of 0.3 nm as extracted from the estimation of the laser pulse numbers, which indicates a Volmer-Weber growth mode of the heteroepitaxy. Considering that the BTO_3-δ_ films are eventually single crystalline, these islands are epitaxial islands, as schematically illustrated in Fig. 3e. We note here that the weak surface interaction due to the Van der Waals force, high mobility, and diffusivity of graphene surface in conjunction with few defect sites serving as nucleation centers (i.e., wrinkles) lead to such Volmer-Weber growth.

Figure 3b shows the surface morphology of the 3 nm-thick BTO_3-δ_ film, which exhibits a much higher island density compared to the 0.3 nm-thick film and BTO_3-δ_ islands almost cover the entire surface. The graphene wrinkles are hardly seen and the RMS is ~1.2 nm. This indicates that the epitaxial island begins to coalesce to form a complete film, as illustrated in Fig. 3f. As the film thickness further increases to 10 nm and 90 nm, the epitaxial BTO_3-δ_ film shows a continuous and flatter surface morphology with the RMS of ~1.3 nm and ~0.8 nm, as shown by AFM images in Fig. 3c, d, respectively. Figure 3g, h shows the schematic illustration of 10 nm and 90 nm thick films, respectively.

It is of significance to explore the strain relaxation of the remote epitaxial BTO_3-δ_ film as a function of the film thickness. XRD measurements were performed on all films (see more details in Fig. S15) and the OP and IP lattice parameters were extracted from the BTO (002) and BTO (200) diffraction peaks, respectively for the strain analysis. Figure 3i shows the evolution of the lattice parameters as a function of the film thickness. For the 3 nm-thick film, the OP and IP lattice parameters are 4.054 Å ± 0.008 Å and 4.024 Å ± 0.009 Å, respectively, which are both larger than those of bulk BTO with tetragonal phase but close to that of cubic BTO. As the film thickness increases, the IP parameter slightly increases while the OP parameter decreases, and they become equal for the 90 nm-thick BTO_3-δ_ film. The evolution of the lattice volume and the c/a ratio as a function of the film thickness, as shown in Fig. 3j, reveals more details. As the film thickness increases, the c/a ratio decreases from 1.006 to 1.000, i.e., the bulk cubic BTO ratio, and the lattice volume slightly decreases from 65.64 Å^3^ to 65.07 Å^3^, which is very close to the bulk cubic BTO volume. These results indicate that the epitaxial BTO_3-δ_ grown on graphene/Ge (011) has a cubic phase due to the ${V}_{O}^{\bullet \bullet }$^35^ induced by the oxygen-poor growth conditions required by the interface engineering. Moreover, different from the BTO/Ge heteroepitaxy^22,33,36^, the BTO_3-δ_ lattice was already partially relaxed at the very beginning of the growth, due to the weakening of the clamping effect of Ge substrates by the graphene monolayer. As the film thickness increases, BTO_3-δ_ relaxes more and becomes fully relaxed in the 90 nm-thick film, while the film becomes less deficiency in oxygen due to a higher oxygen partial pressure during the growth, and thus the lattice volume decreases^37^. Such an observation expands the understanding of the mechanism of heteroepitaxy, particularly, including two-dimensional materials.

### Flexible membranes

**Fig. 4:**
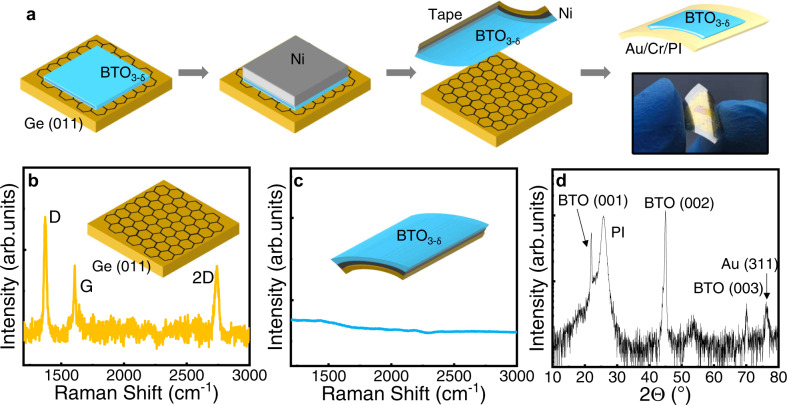
Exfoliation and transfer of the BTO_3-δ_ film on a flexible PI substrate. **a** Schematic illustration of the BTO_3-δ_ film growth, exfoliation, and transfer process onto a flexible PI substrate. Raman spectra of **b** the Ge (011) surface and **c** the exfoliated film surface. **d** Specular XRD pattern of the BTO_3-δ_ film transferred onto a flexible PI substrate.

Considering that the van der Waals force existing at the interface of BTO_3-δ_/graphene/Ge is rather weak, the potential of the exfoliation of remote epitaxial BTO_3-δ_ films was investigated and the results were shown in Fig. 4. Figure 4a illustrated the whole process, which includes firstly the deposition of a 1 μm-thick Ni stressor layer, then the exfoliation using a thermal release tape and finally the transfer of the BTO_3-δ_ film on arbitrary substrates, e.g., flexible polymer substrates (in this case, PI) and Si substrates etc. The successful exfoliation of the BTO_3-δ_ film arises from the much stronger bonding force of the Ni/BTO_3-δ_ interface compared to that of the BTO_3-δ_/graphene/Ge interface. It is noted here that the growth conditions of Ni stressor layers were carefully optimized to provide sufficient tensile stress (see Methods section for more details). Raman spectroscopy was carried out on the Ge substrate and the exfoliated layers to examine the location of the graphene monolayer, and the spectra were shown in Fig. 4b and c, respectively. It can be seen that the Raman spectrum of Ge (011) substrate shows three vibration modes at 1372 cm^−1^, 1608 cm^−1^, and 2740 cm^−1^, corresponding to the D, G, and 2D peaks of the graphene single layer, respectively, which indicates that the graphene remains on the Ge substrate after  the exfoliation. The appearance of the D peak suggests some defects in graphene, possibly formed during the growth or the exfoliation process. Moreover, compared to the pure clean graphene monolayer, the G and 2D peaks undergo a blue shift, revealing possible oxidation of the graphene^38,39^. The Raman spectrum of the exfoliated BTO_3-δ_/Ni layers, as shown in Fig. 4c, reveals no peak in the range of 1400–3000 cm^−1^, indicating of the absence of graphene. Scanning electron microscopy (SEM) characterization was also performed on Ge (011) and BTO_3-δ_ layer after the  exfoliation, accompanied with energy dispersive X-ray spectroscopy (EDS) analysis, and the results are shown in Fig. S16, which shows a flat morphology of both surfaces.

The remote epitaxial 90 nm-thick BTO_3-δ_ films were transferred on flexible PI and Si substrates, respectively. Figure 4d shows the specular 2*θ* XRD scan of the BTO_3-δ_/Au/Cr/PI stack, where the BTO (001), (002), and (003) reflections can be clearly identified at 2*θ* = 22.00°, 44.97°, and 70.11°, respectively. This further corroborates the good single crystallinity of the BTO_3-δ_ film on graphene/Ge (011). The extracted OP lattice parameter of the 90-nm thick BTO_3-δ_ on PI is ~4.028 Å, larger than the as-grown one, which could result from the release/absence of strain in the as-grown film due to the substrate clamping. The XRD pattern of BTO_3-δ_ on Au/Cr/SiO_2_/Si substrates shows similar single crystallinity of the BTO_3-δ_ film, more details of which can be found in Fig. S17 in SI.

### Flexoelectric property of exfoliated BTO_3-δ_ thin films

**Fig. 5:**
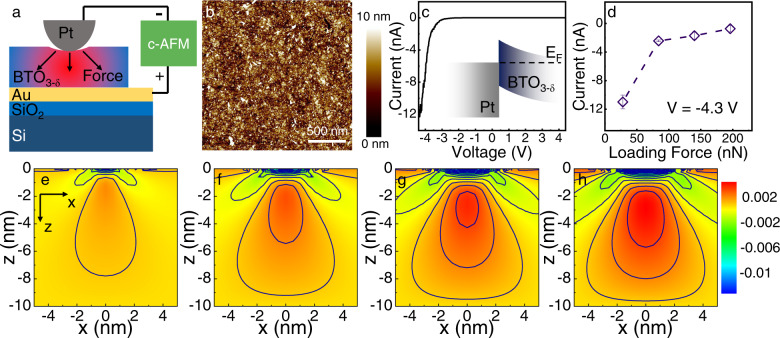
Enhanced flexoelectric properties of the BTO_3-δ_ membrane. **a** Illustration of the experimental configuration of the c-AFM test. **b** AFM image of the transferred BTO_3-δ_ film. **c** *I*–*V* curve of the Pt/BTO_3-δ_ junction. **d** Evolution of the current as a function of loading force. **e**–**h** COMSOL FEM calculation with a tip-force model of the BTO_3-δ_ film under applied force of 28 nN, 84 nN, 140 nN, and 196 nN, respectively. The colors of blue to red correspond to lower to higher strain values. The solid lines in the figures represent the equivalent strain value profile.

Flexoelectric effect is an effect widely existing in solid materials, including dielectric, semiconductor, conductor and two-dimensional materials, especially for ultra-thin materials (which can produce greater deflection). Considering that functional oxide thin films are of great potential for many applications like flexible devices, the flexoelectricity of the transferred BTO_3-δ_ films was examined by using conductive-AFM (c-AFM). Figure 5a shows the measurement configuration, where the Pt tip of the c-AFM cantilever forms a contact with the exfoliated and transferred 10 nm-thick BTO_3-δ_ film on the Au/Cr/SiO_2_/Si substrate. Figure 5b shows the surface morphology of the BTO_3-δ_ film, with an RMS of 1.9 nm, which is only slightly higher than that of the as-grown film (1.3 nm, Fig. 3c). Figure 5c exhibits the typical *I–V* curve obtained from the Pt / BTO_3-δ_/Au stack, indicating a rectifying behavior with a turn-on voltage of about −2.4 V. This suggests that the BTO_3-δ_ is n-type semiconducting and forms a Schottky junction with the Pt electrode. Figure 5c inset illustrates the band structure of this heterostructure. By applying different loading force on the BTO_3-δ_ film using the AFM tip, the strain gradient and the polarization in the film would be changed and thus lead to the modification of the Schottky barrier height and subsequently the variation of the average current flowing through the BTO_3-δ_ film, which can be detected by c-AFM, as shown in Fig. 5d. Here the applied AFM tip bias was set to −4.3 V and the current of each contact point was measured for three times. It can be evidently observed that, as the loading force increases from 28 nN, to 84 nN, to 140 nN, and to 196 nN, the current varies from ~−12 nA to −1 nA. To evaluate the strain distribution of the exfoliated BTO_3-δ_ film, particularly under different applied forces ranging from 28 nN to 196 nN, COMSOL finite element analysis was performed and the results were shown in Fig. 5e–h (see Method section for more details), in which the blue region on the film surface represents the AFM tip. It can be seen that the high strain value region (red) appears right under the AFM tip and diffuses deeper into the BTO_3-δ_ film bulk (toward the interface at z = −10 nm) as the applied force increases. The gauge factor (GF) of the BTO_3-δ_ film can be calculated using the formula:

$${{{{{\rm{GF}}}}}}=(\Delta I/I)/\Delta \varepsilon$$

where *I* and *ε* represent current and strain, respectively^40^. The GF of the exfoliated BTO_3-δ_ film reaches 1127.

The GF is defined by the change of current under mechanical stress. Both piezoelectric effect and flexoelectric effect will cause the change of polarization under mechanical stress, resulting in the change of current. However, for our BTO_3-δ_ films with the cubic phase at room temperature, no piezoelectric effect was found using piezoresponse microscopy (PFM) measurements, as shown in Fig. S18 in SI. Therefore, the GF of paraelectric cubic BTO_3-δ_ films is impacted only by the flexoelectric effect.

Flexoelectric effect is the change of polarization caused by strain gradient. Generally, more obvious flexoelectric effect can be obtained in thin films than in bulk materials^4^. Table S1 lists the flexoelectric properties of oxides in the form of single crystal, ceramic and thin films. Compared with GFs of commonly studied Nb-doped STO (Nb:STO) (183)^40^, the GF value of our BTO_3-δ_ film (1127) is much larger. Moreover, we note here that the GF of a 10 nm-thick stochiometric BTO film with tetragonal phase is only 235. Therefore, the BTO_3-δ_ exfoliated remote epitaxial film has a GF being four times larger than that of “conventional” BTO films. The stoichiometric epitaxial BTO film was fabricated on Nb:STO using PLD in oxygen-rich ambience at 650 °C, which was reported by many groups^34,41,42^. XRD RSM patterns shown in Fig. S20 and PFM domain patterns shown in Fig. S21 indicate the tetragonal phase and the good piezoelectric properties of such stoichiometric BTO films. The GFs of BTO films on Nb:STO were measured using a similar method by c-AFM (*V* = −1.5 V and AFM tip force of 28 nN to 196 nN). More details can be found in Fig. S22 in SI.

Let us now consider the possible origin of the excellent flexoelectric property of the exfoliated remote epitaxial BTO_3-δ_ films. The flexoelectric property is related to the strain gradient and the polarization of the material. We believe that the free-standing characteristic and the ${V}_{O}^{\bullet \bullet }$ induced phase transition, as well as the polarization variation, are the main reasons. First, the exfoliated films have no chemical bonding with the substrate and thus have much less clamping effect, which leads to the enhanced strain gradient response under applied stress. Secondly, BTO_3-δ_ possesses cubic phase, which has very different mechanical properties (e.g., Young’s modulus and Poisson’s ratio) compared with its tetragonal phase, which makes the strain gradient be strongly modified. Thirdly, as reported by ref. ^28,43^. and our results, ${V}_{O}^{\bullet \bullet }$ make the BTO_3-δ_ be semiconducting with larger permittivity, which has a direct proportional relationship with the flexoelectric coefficient of BTO^44,45^.

In order to further understand the flexoelectric properties of the BTO_3-δ_ films, the effect of thickness of BTO_3-δ_ films was studied. The exfoliated and transferred 90 nm-thick BTO_3-δ_ films were measured using c-AFM. However, the detected current was rather weak with the applied force range (28–196 nN). FEM calculation reveals that it is probably because the strain gradient occurs only close to the film surface area whereas the film bulk remains intact. More details can be seen in Fig. S24. In a 90 nm-thick film, taking 196 nN force as an example, the calculated maximum strain value is 0.00260, which is less than that of the 10 nm-thick film of 0.00323. Therefore, GF decreases sharply as the film thickness increases.

In conclusion, single-crystalline flexoelectric BTO_3-δ_ membranes have been fabricated using remote epitaxy on graphene-passivated Ge substrates. DFT ab initio calculations suggested a higher potential fluctuation of Ge (011) than Ge (001). Such difference was confirmed by the different crystallographic properties of BTO_3-δ_ films grown on graphene/Ge (001) and graphene/Ge (011) substrates, respectively. Detailed XRD and TEM investigations demonstrated that the remote epitaxy of single-crystalline BTO_3-δ_ films was realized on Ge (011) substrate, whereas the BTO_3-δ_ films grown on graphene/Ge (001) turned out to be textured with a (001) orientation. Both heterostructures show sharp interfaces, indicating the passivation role of the graphene monolayer, which is of great importance for highly heterogenous oxide/semiconductor systems. The remote epitaxy of BTO_3-δ_ followed a Volmer-Weber 3D growth mode and the film was already partially relaxed at the very initial stage of the growth, due to the weakening of the clamping effect from the substrate by the graphene layer. The BTO_3-δ_ films can be exfoliated and transferred to arbitrary substrates like Si and PI using a Ni stressor layer, with the graphene layer remaining on the Ge substrate after exfoliation. The oxygen vacancies in BTO led to a semiconducting cubic phase of BTO_3-δ_ and enhanced its flexoelectric property with a gauge factor of as high as 1127. Our results deepen the understanding of the fundamental physics of remote epitaxy for highly heterogeneous oxide/semiconductor systems and demonstrate a great potential of such BTO_3-δ_ films for functional devices, particularly the wearable and flexible devices.

## Methods

### DFT calculations

DFT calculations were performed using the Vienna Ab Initio Simulation Package^46–48^. The plane-wave cutoff was set to 400 eV based on test calculations. The Perdew-Burke Ernzerhof exchange-correlation functional for solids (PBEsol)^49^ was used in combination with the projector-augmented wave (PAW)^50^ method that describes the interaction between core ions and valence electrons.

As the PBEsol-optimization results of lattice constant of bulk Ge are quite close to the experimental value (*a*_0_ = 5.658 Å for the standard diamond unit cell), we constructed slab models for both Ge (001) and (011) surface directly based on the experimental bulk structure of Ge. The Ge (001) surface model was constructed using an orthogonal cell with dimensions $a=\sqrt{2}{a}_{0}$ and $b={a}_{0}/\sqrt{2}$ along the [110] and $\left[\bar{1}10\right]$ directions of the Ge (diamond) structure, respectively (the ${Fd}\bar{3}m$ symmetry allows to construct a slab model with periodic boundary conditions in this way). The slab model consists of 24 Ge atoms in 12 layers, where the central 4 layers were kept frozen to mimic the Ge bulk and all other (i.e., surface) Ge atoms were relaxed in all calculations. For the Ge (011) surface, an orthogonal slab model was constructed with $a=3{a}_{0}$ and $b={a}_{0}/\sqrt{2}$ along the [100] and $\left[01\bar{1}\right]$ directions of the Ge bulk, respectively. It consists of 36 Ge atoms in 6 layers, where the geometry of the central 2 layers was fixed in DFT calculations. On each surface, the third dimension of the slab model was specified according to the varying thickness of vacuum space, in particular a 4.8 nm-thick vacuum layer was used together with surface-dipole correction to minimize the inter-slab interaction. Γ-centered 4 × 8 × 1 and 2 × 8 × 1 *k*-point meshes were used for the Brillouin-zone integrations for Ge (001) and Ge (011) surface models, respectively.

A graphene monolayer was initially placed a few Å above the Ge (001) surface with its $\left[2\bar{1}\bar{1}0\right]$ direction along *a* and $\left[01\bar{1}0\right]$ along *b* of the Ge (001) slab model. Similarly, a monolayer of graphene was inserted into the vacuum with its $\left[2\bar{1}\bar{1}0\right]$ direction along *a* and $\left[01\bar{1}0\right]$ along *b*. The positions of all C atoms were optimized so that the graphene-Ge distances were evaluated based on the relaxed graphene/Ge structures for both Ge surface models.

### Graphene growth and detection

The graphene was grown on Ge substrates using CVD. More details can be found in our prior publications of ref. ^29,51^. The graphene layer properties were characterized by Raman spectroscopy using a LabRAM HR Evolution (Horiba Scientific) system (the laser wavelength of 532 nm and grating lines of 600).

### Pre-treatment and thin-film growth

A pre-treatment was carried out on the graphene/Ge substrate prior to the film deposition. The substrate was heated to 650 °C in the PLD chamber at the base pressure of <1 × 10^−4 ^Pa and such a pre-treatment continued for 10 minutes to remove the possible surface contamination on the graphene. The stoichiometric BTO ceramic target was placed ~4.5 cm away from the substrate. A 248 nm laser with a frequency of 2 Hz was used to excite the plume. The targeting thicknesses of the samples were estimated using the number of laser pulses. The equivalent thickness of a single laser pulse is ~0.03 nm. After the film growth, the sample was cooled down to the room temperature at the base pressure. During the whole process, the oxygen partial pressure was kept below 5 × 10^−4 ^Pa.

### Morphology and crystallographic

Atomic force microscopy (Dimension Icon, Bruker) was used to examine the surface morphology of the BTO_3-δ_ thin films. The AFM was operated at ScanAsyst mode using a SCANASYST-AIR probe. Each area was scanned with 512 lines and the scan rate was 0.5 Hz.

X-ray diffraction (SmartLab, Rigaku) was used to study the crystallinity of the BTO_3-δ_ films. The wavelength of the X-ray was 1.54056 Å which is generated by a Cu-target X-ray tube. The parallel beam was monochromatized using a Ge (220) × 2-bounce monochromator.

Transmission electron microscopy (Tecnai, FEI) operating at 200 kV was used to examine the cross-section of the samples, and also the crystallinity of the BTO_3-δ_ films. The TEM specimen was fabricated using a focused ion beam (FIB, Scios, FEI) system.

### Membrane exfoliation and transfer

To exfoliate the BTO_3-δ_ films grown on Ge via a graphene monolayer, a Ni stressor layer was fabricated on the BTO_3-δ_ film surface. A 30 nm-thick Ni layer was evaporated onto the BTO_3-δ_ using electron beam evaporation (TF500, HHV Ltd.) and then a 1-μm thick Ni was sputtered using a magnetron sputtering (JGP560D, SKY Co., Ltd). The whole stack was stuck to a thermal release tape, which released the Ni stressor and the BTO_3-δ_ film stack to the targeting substrate. The Ni stressor was then dissolved by FeCl_3_.

### COMSOL simulation

To investigate the distribution of strain of the thin films, simulation was carried out using COMSOL Multiphysics. The Pt probe and the surface of the thin film were set to contact using Hertz model^40^. The radius of the probe was ~25 nm. The detailed parameters used in the process of the simulation can be found in SI.

### Reporting summary

Further information on research design is available in the Nature Research Reporting Summary linked to this article.

## Supplementary information

Supporting Information

Reporting Summary

## Data Availability

The datasets generated during and/or analyzed during the current study are available from the corresponding author on reasonable request.
