# Supplementary material for: Highly heterogeneous epitaxy of flexoelectric BaTiO3-δ membrane on Ge
Source: Nat Commun. 2022 May 30;13:2990. doi: 10.1038/s41467-022-30724-7 (PMC9151678; doi:10.1038/s41467-022-30724-7)
Supplement: Supplementary file 1 — Supporting Information [file 41467_2022_30724_MOESM1_ESM.pdf]

Supporting Information

Highly Heterogeneous Epitaxy of Flexoelectric $BaTiO_{3-\delta}$ Membrane on Ge

Liyan Dai,[1] Jinyan Zhao,[1] Jingrui Li,[1*] Bohan Chen,[1] Shijie Zhai,[1] Zhongying Xue,[2] Zengfeng Di,[2] Boyuan Feng,[3] Yanxiao Sun,[1] Yunyun Luo,[4] Ming Ma,[1] Jie Zhang,[1] Sunan Ding,[3] Libo Zhao,[4] Zhuangde Jiang,[4] Wenbo Luo,[5] Yi Quan,[1,6] Jutta Schwarzkopf, [7] Thomas Schroeder, [7] Zuo-Guang Ye,[8] Ya-Hong Xie, [9] Wei Ren[1*] and Gang Niu[1*]

[1]. Electronic Materials Research Laboratory, Key Laboratory of the Ministry of Education & International Center for Dielectric Research, School of Electronic Science and Engineering & The International Joint Laboratory for Micro/Nano Manufacturing and Measurement Technology, Xi'an Jiaotong University, Xi'an 710049, China

*E-mail: gangniu@xjtu.edu.cn; jingrui.li@xjtu.edu.cn; wren@xjtu.edu.cn

[2]. State Key Laboratory of Functional Materials for Informatics, Shanghai Institute of Microsystem and Information Technology, Chinese Academy of Science, Shanghai, 200050, China

[3]. Suzhou Institute of Nano-Tech and Nano-Bionics, Chinese Academy of Sciences, Suzhou, 215123, China

[4]. The State Key Laboratory for Manufacturing Systems Engineering & The International Joint Laboratory for Micro/Nano Manufacturing and Measurement Technology, Xi'an Jiaotong University, Xi'an 710049, China

[5]. State Key Laboratory of Electronic Thin Films and Integrated Devices, University of Electronic Science and Technology of China, Chengdu, 611731, China

[6]. School of Microelectronics, Xidian University, Xi’an, 710071, China

[7]. Leibniz-Institut für Kristallzüchtung, Max-Born-Straße 2, Berlin, 12489, Germany.

[8]. Department of Chemistry and 4D LABS, Simon Fraser University, Burnaby, British

Columbia, V5A 1S6, Canada

[9]. Department of Materials Science and Engineering, University of California, Los Angeles, California, 90095, USA

1. Graphene/Ge substrate

High quality graphene were grown on Ge substrates by chemical vapor deposition (CVD).[1,2] The surface morphology of the sample is shown in Figure S1. The root mean square (RMS) roughness of the graphene/Ge (001) and graphene/Ge (011) surface is 2.8 nm and 0.3 nm, respectively. In particular, the wrinkle of graphene can be seen on the surface of graphene/Ge (011) (Figure S1 (b)). The G peak and 2D peak of graphene can be seen by Raman spectroscopy.

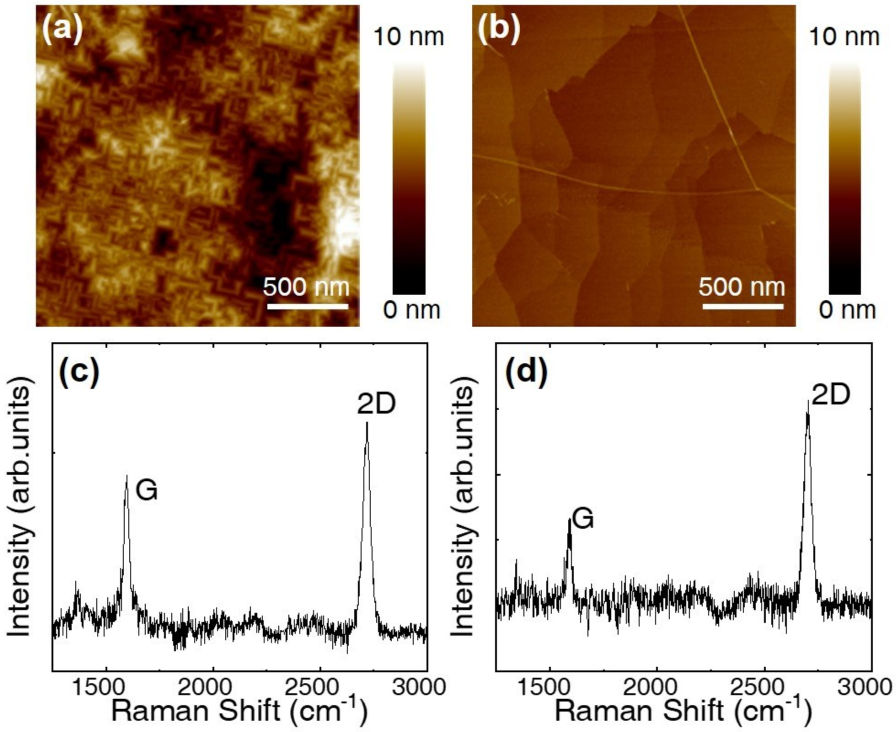

Figure S1 (a) AFM image of as-received graphene/Ge (001) substrate. (b) AFM image of as-received graphene/Ge (011) substrate. (c) Raman spectrum of as-received graphene/Ge (001) substrate. (d) Raman spectrum of as-received graphene/Ge (011) substrate.

2. Potential fluctuation through graphene

Figure S2 schematically shows the epitaxial growth of BTO on Ge, where the gap distances of graphene to Ge substrate and to BTO film are labeled as ‘i’ and ‘ii’, respectively. The value of ‘i’ is about 0.35 nm for Ge (001) substrate or 0.36 nm for Ge (011) substrate based on ab initio DFT calculations. Adopting about 0.35 nm for ‘ii’

according to Ref. 3, we calculate the potential fluctuation at z = 0.7 nm away from Ge surface, which is the approximate distance between the grown BTO film and Ge with a monolayer graphene in between.

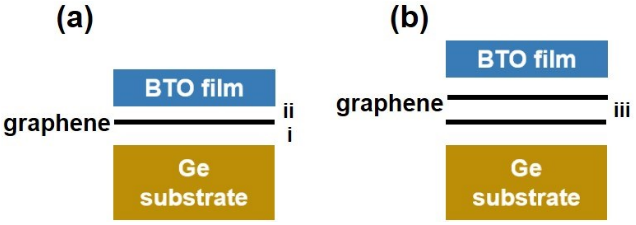

Figure S2 Sketches of the layered Ge/graphene/BTO models with (a) BTO film on monolayer graphene on Ge substrate, and (b) BTO film on bilayer graphene on Ge substrate.

We further calculate the potential fluctuation at z = 1.0 nm, as this is the approximate BTO – Ge distance when inserting two graphene layers between them, as the distance between graphene layers is 0.335 nm (marked by iii) according to the prior reports.[4] As it shown in Figure S3, the potential fluctuation at z = 1.0 nm away from the Ge surface is much smaller than the fluctuation of Ge (001) through monolayer graphene. This indicates that the remote epitaxy of BTO would be difficult to be realized through bilayer graphene for both (001) and (011) oriented Ge.

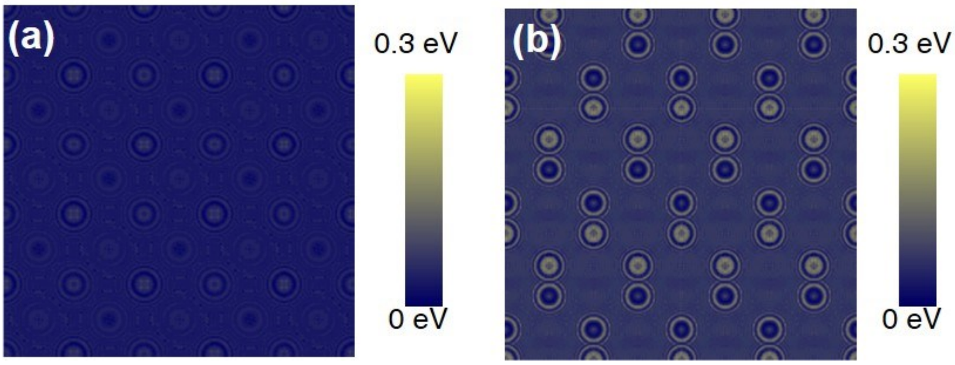

Figure S3 DFT-calculated potential fluctuation for the Ge substrate (1.0 nm above the surface). (a) for the Ge (001) substrate (b) for the Ge (011) substrate.

The screening effect of graphene on Ge substrate has also been investigated. The models are similar to Figure 1 (c) and (d) but with a monolayer graphene inserted at 0.35 nm above the Ge surface. Figure S4 shows that, at z = 0.7 nm, the potential

fluctuation of Ge (011) is more obvious than that of Ge (001) which is also true for models without graphene (Figure 1 (c) and (d)). For each surface, the fluctuation map exhibits noticeable correlation with the locations of surface Ge atoms in a way similar to Figure 1 (c) and (d), which is superimposed by some contribution from the graphene carbon atoms. The overall screening effect of graphene on the potential fluctuation and thus the epitaxial growth of BTO is relatively weak.

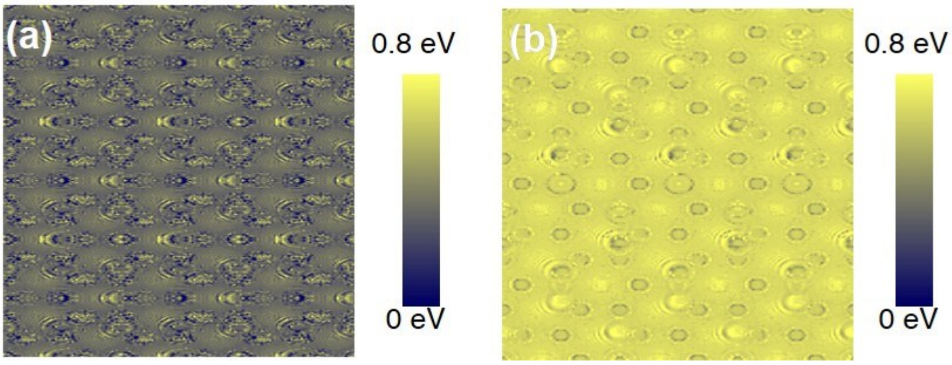

Figure S4 DFT-calculated potential fluctuation for the Ge substrate (0.7 nm above the surface) covered by monolayer graphene at 0.35 nm above the Ge surface. (a) for the Ge (001) substrate (b) for the Ge (011) substrate.

3. Pretreatment of graphene/Ge substrate

After pretreatment in the vacuum chamber of pulsed laser deposition (PLD), it can be seen that the RMS roughness of graphene/Ge substrate decreased to 2.2 nm and 0.2 nm, respectively.

The graphene/Ge (011) surface have holes when the substrate maintained 650 ℃ with the introduction of oxygen (P~2 Pa) into the PLD chamber, as shown in Figure S6 (a) and (b) by optical microscopy (OM) and scanning electron microscopy (SEM), respectively. The SEM photo shown Figure S6 (b) reveals that holes in Ge in fact have facets due to the anisotropy of the etching rate.

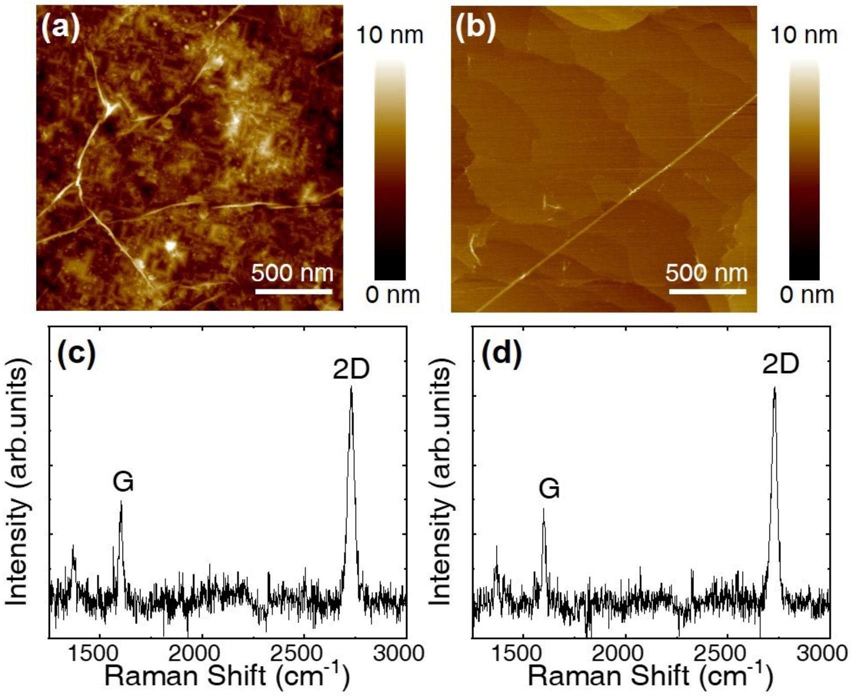

Figure S5 (a) AFM image after pretreatment of graphene/Ge (001) substrate. (b) AFM image after pretreatment of graphene/Ge (011) substrate. (c) Raman spectrum after pretreatment of graphene/Ge (001) substrate. (d) Raman spectrum after pretreatment of graphene/Ge (011) substrate.

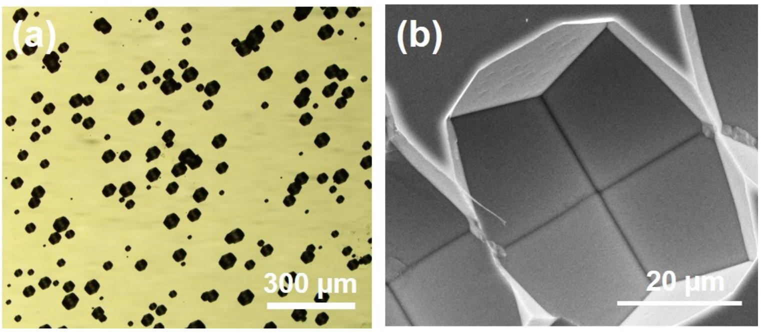

Figure S6 (a) Optical microscope photos of graphene / Ge (011) substrate after oxidation. (b) SEM photos of graphene / Ge (011) substrate after oxidation.

4. Crystallinity of BTO thin films on Ge through monolayer graphene

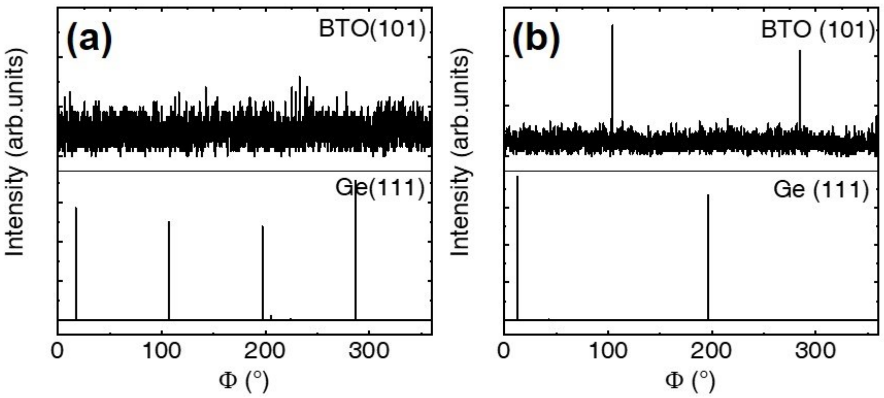

Figure S7 (a) $BTO_{3-\delta}$/graphene/Ge (001) Φ scan of BTO (101) peak (upper) Ge (111) peak (lower). (b) $BTO_{3-\delta}$/graphene/Ge (011) Φ scan of BTO (101) peak (upper) Ge (111) peak (lower).

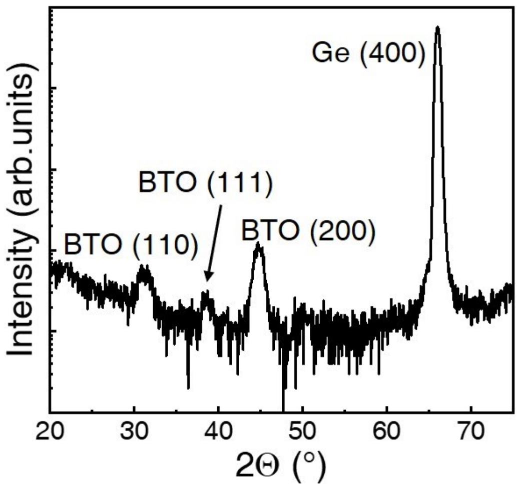

Figure S8 In-plane XRD patterns of BTO/graphene/Ge (001) films.

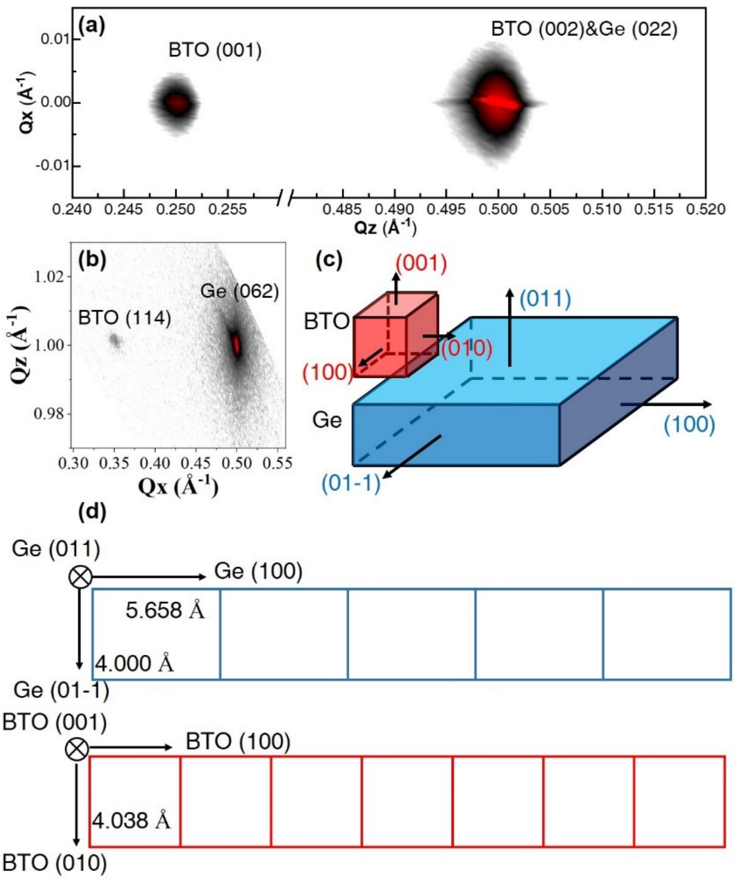

Figure S9 (a) $BTO_{3-\delta}$/graphene/Ge (011) symmetry plane RSM. (b) $BTO_{3-\delta}$/graphene/Ge (011) asymmetry plane RSM. (c) Epitaxial relationship of the BTO film and Ge (011) substrate. (d) lattice parameters of the Ge substrate and the BTO film.

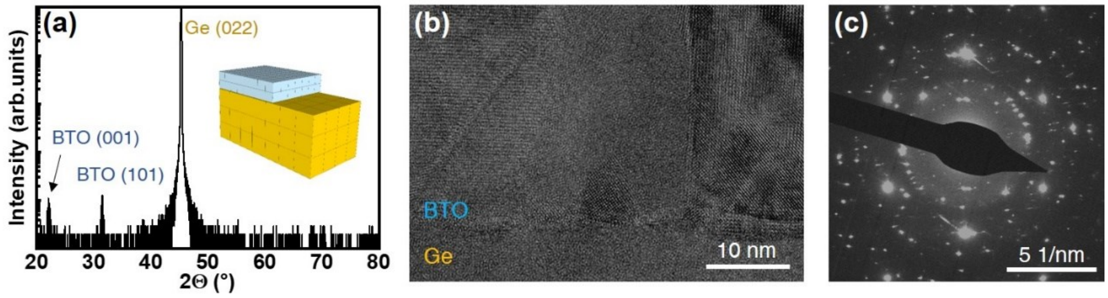

Figure S10 (a) Out-of-plane XRD patterns of $BTO_{3-\delta}$ films grown on Ge (011). (b) Cross-section TEM image of the $BTO_{3-\delta}$/Ge (011). (c) SAED pattern of the $BTO_{3-\delta}$/Ge (011).

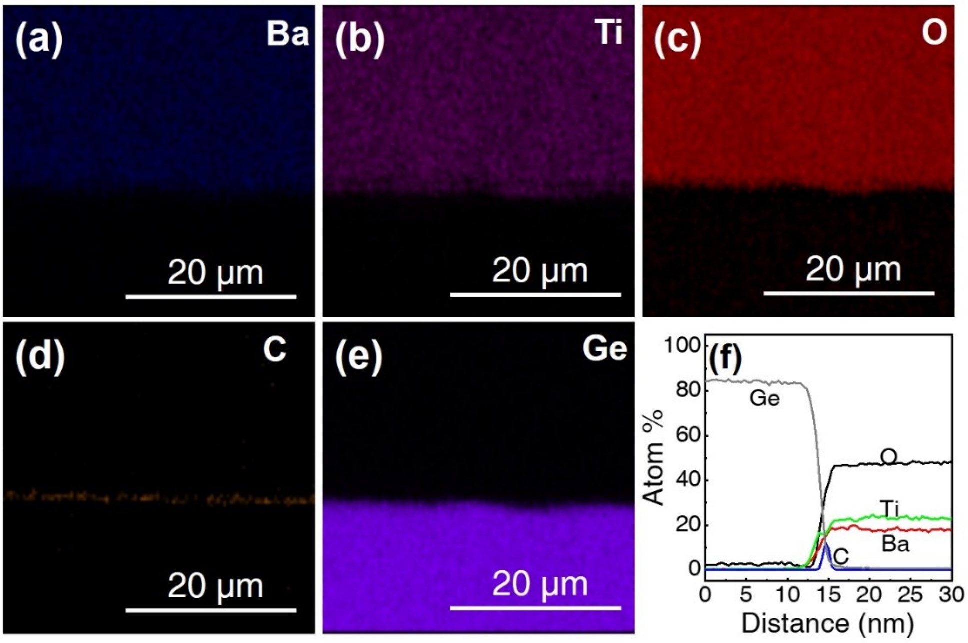

Figure S11 EDX mappings of the $BTO_{3-\delta}$ thin film shown in Figure 2 (i). From (a) to (e), mappings of the Ba, Ti, O, C and Ge elements, respectively. (f) EDX line profile of the interface.

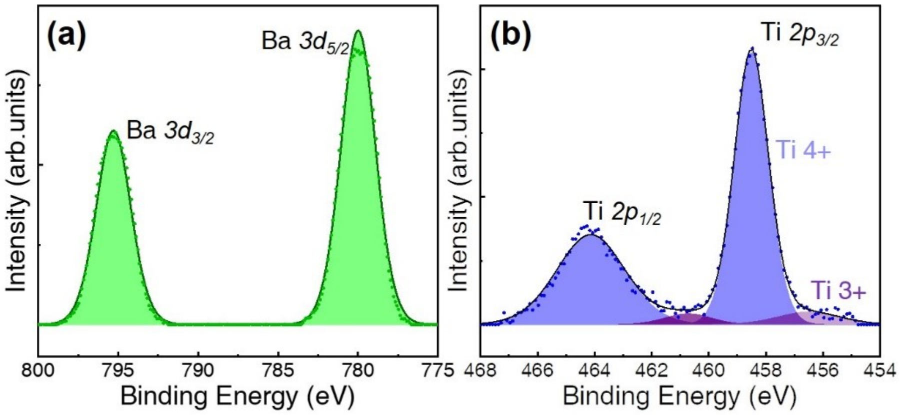

Figure S12 XPS results of the remote epitaxial $BTO_{3-\delta}$ thin film. (a) Ba, (b) Ti, respectively. The dots are experimental data and the lines are the fitted profile, while the filling blocks are the deconvoluted peaks, in which the peak pair at lower BE in dark purple represents Ti^{3+} component while the peak pair at higher BE in blue represents Ti^{4+} component.

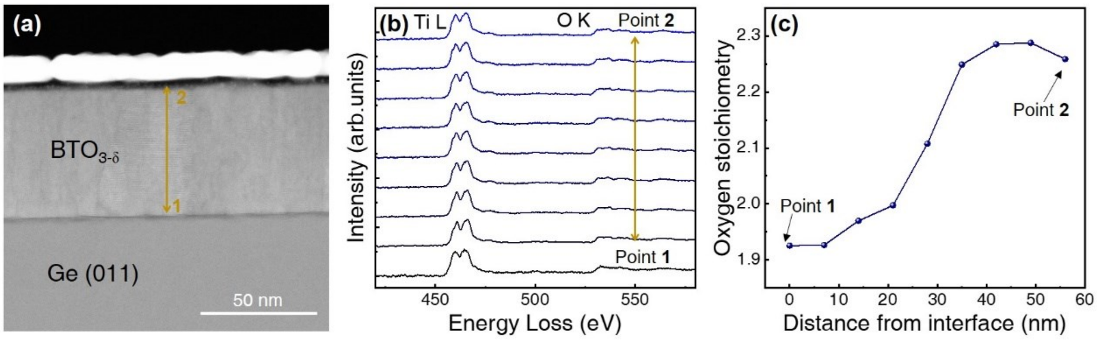

Figure S 13 (a) cross sectional STEM image of the BTO$_{3-\delta}$ /graphene/Ge (011) thin film. (b) EELS on the BTO$_{3-\delta}$ thin film. (c) oxygen stoichiometry (O/Ti ratio) of the BTO$_{3-\delta}$ thin film across the film thickness.

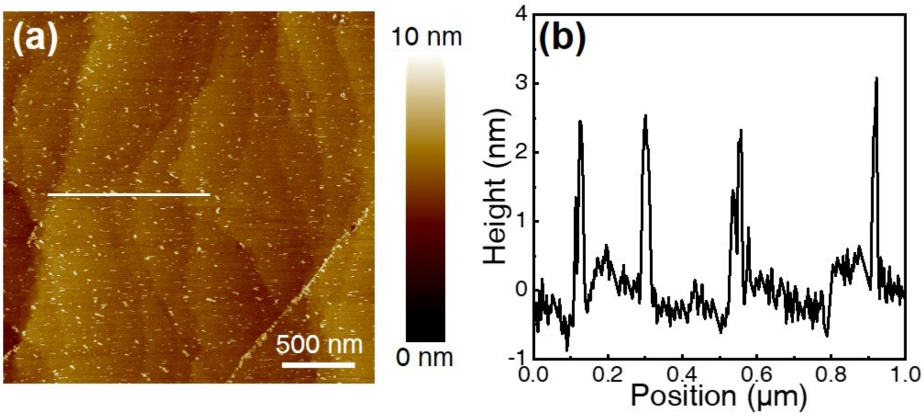

Figure S14 (a) AFM image from Figure 3 (a) and (b) line profile of the AFM images. The average height of the islands is about 2.5 nm.

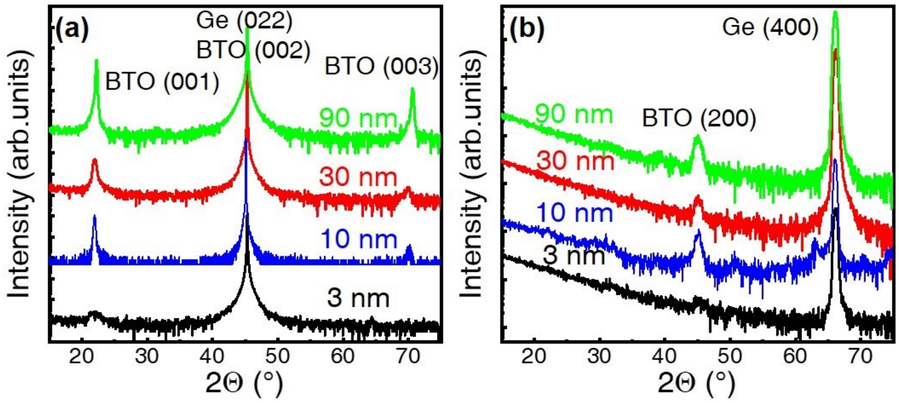

Figure S15 (a) Out-of-plane XRD patterns of BTO$_{3-\delta}$/graphene/Ge (011) films with different thicknesses by remote epitaxy. (b) In-plane XRD patterns of BTO$_{3-\delta}$/graphene/Ge (011) films with different thicknesses by remote epitaxy.

5. Exfoliation and transfer of the $BTO_{3-\delta}$ film

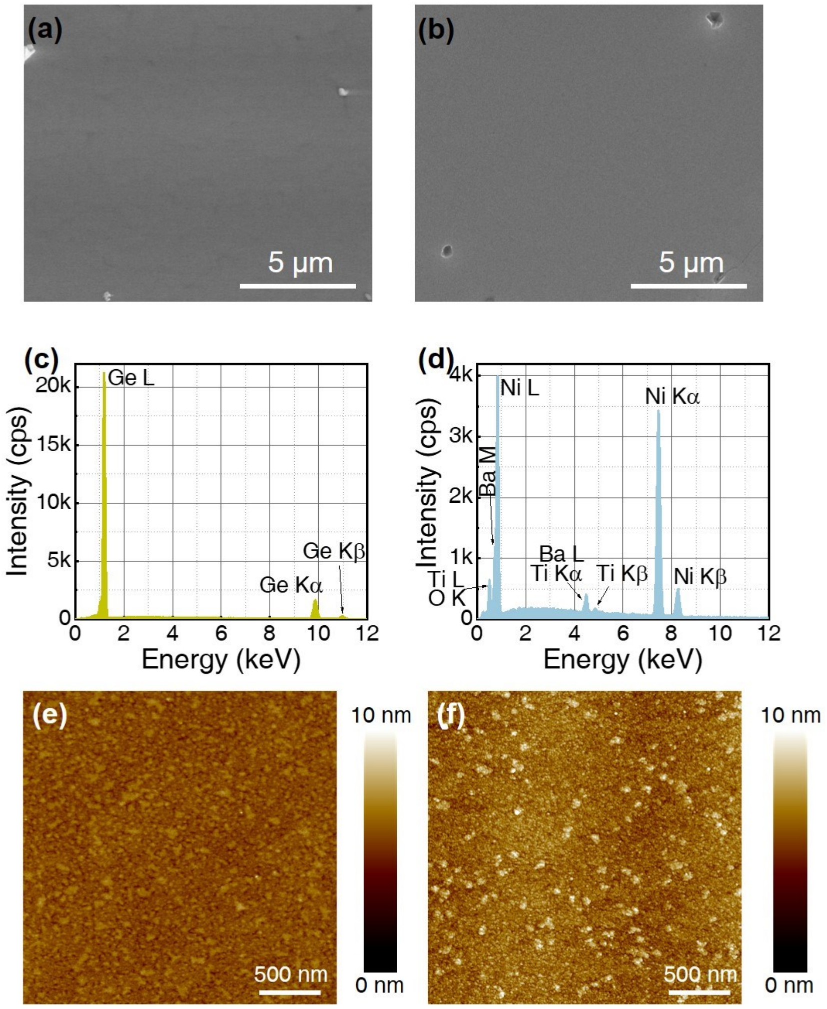

Figure S16 After remote epitaxial film exfoliation, (a) SEM photo of remaining substrate. (b) SEM photo of exfoliated film. (c) Energy spectrum of remaining substrate. (d) Energy spectrum of exfoliated film. (e) AFM image of remaining substrate. (f) AFM image of exfoliated film.

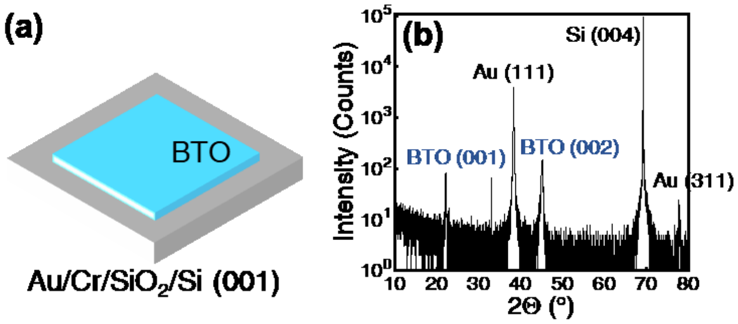

Figure S17 (a) Schematic figure for the heterostructure after BTO being transferred to Si substrate. (b) corresponding OP XRD pattern.

6. Electrical characterization of the $BTO_{3-\delta}$ thin film

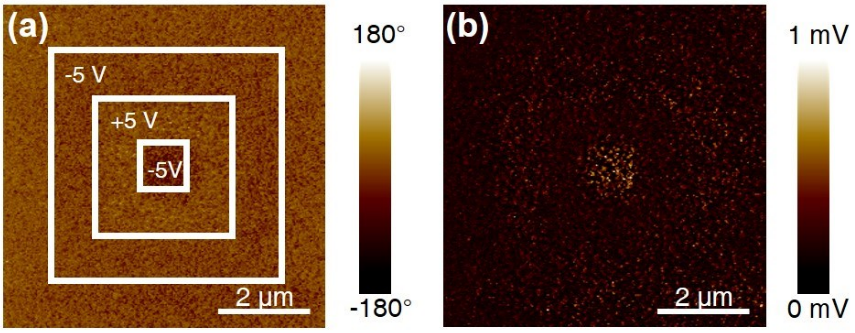

Figure S18 (a) PFM phase diagram of the $BTO_{3-\delta}$ thin film transferred to Si substrate. (b) PFM amplitude diagram of the $BTO_{3-\delta}$ thin film transferred to Si substrate.

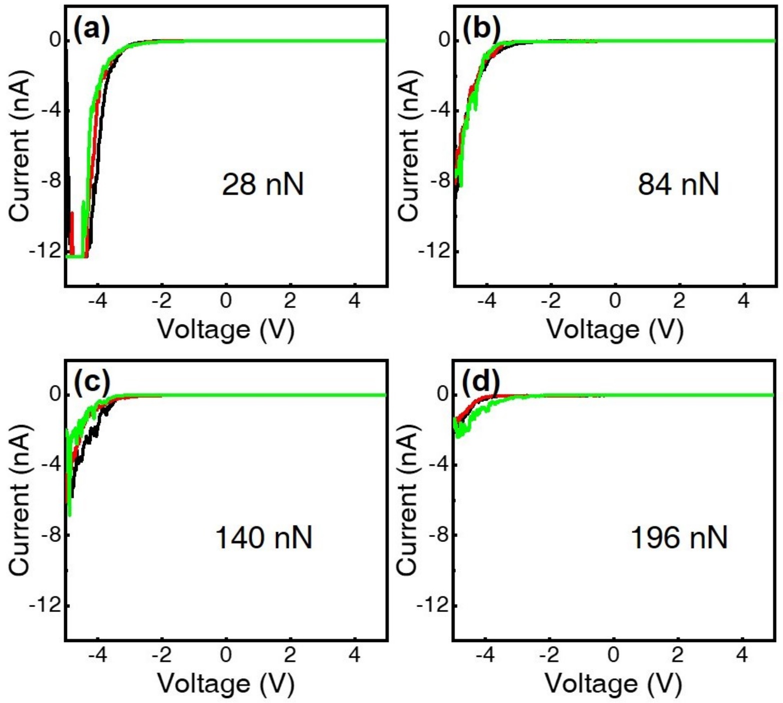

Figure S19 (a) to (d) *I-V* curve of the Pt/$BTO_{3-\delta}$ junction with a tip-force of the $BTO_{3-\delta}$ film under applied force of 28 nN, 84 nN, 140 nN and 196 nN, respectively. Red, yellow and black line refer to repeating measurements (three times) of the same conditions.

7. Flexoelectricity of BTO thin films

In order to clarify the origin of the large GF of 1127 of the $BTO_{3-\delta}$ thin films, a 10 nm-thick stoichiometric BTO thin film was grown on Nb:STO substrate by PLD using the oxygen rich ambience at 650°C, which has the tetragonal phase and has the

ferroelectric property at room temperature.[5–7] The crystallinity of the BTO thin film was examined using RSM which was shown in Figure S20. The out-of-plane and in-plane lattice parameters extracted from RSM were 4.120 Å and 3.909 Å respectively. The piezo-(ferro-)electric properties were verified using PFM and the results are shown in Figure S21. Three concentric boxes were "written" on the BTO thin film with a voltage of -5/+5/-5 V, and the polarization state was "read" with a voltage of 800 mV after 3 hours.

We investigated such a "conventional" BTO film using the c-AFM with exactly the same setup and parameters with the $BTO_{3-\delta}$ films and the *I-V* curves were shown in Figure S22. Different from the transferred semiconducting $BTO_{3-\delta}$ films, the piezoelectric film directly grown on the conductive Nb:STO substrate has much greater current at the same applied voltage. When $V = -1.5$ V, the currents with 28nN, 84nN, 140nN and 196nN applied tip forces were shown in Figure S22 (a) to (d), respectively. The average value was obtained through three measurements. The variation of the current as a function of loading force was shown in Figure S22 (e). As the applied force increases from 28nN to 196nN, the detected c-AFM current is always about -2 nA ~ -3 nA. The simulation of the strain generated on the BTO film was shown in Figure S23, and the GF was calculated to be about 235. The exfoliated remote epitaxial $BTO_{3-\delta}$ film has a GF 4 times larger than the conventional BTO films.

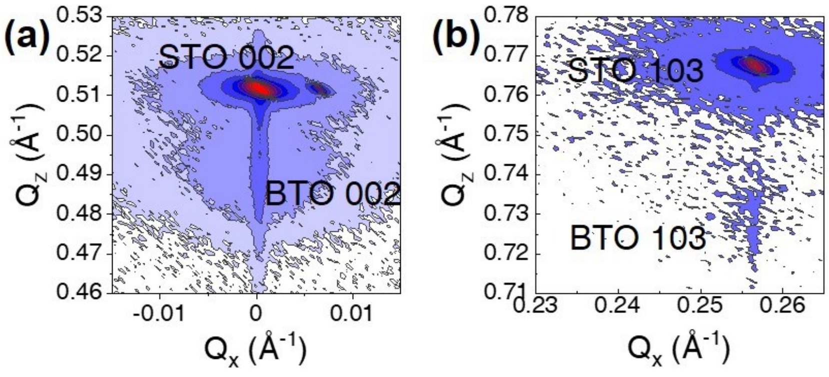

Figure S20 (a) RSM of BTO (002) and STO (002). (b) RSM of BTO (103) and STO (103).

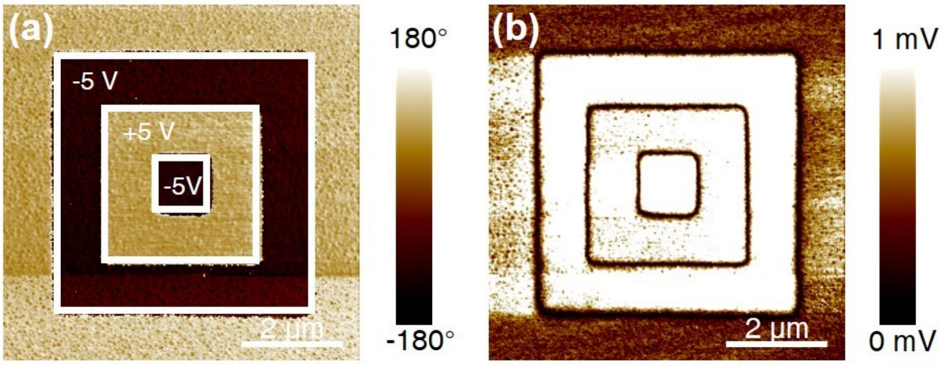

Figure S21 (a) PFM phase diagram of the BTO thin film grown on Nb:STO substrate. (b) PFM amplitude diagram of the BTO thin film grown on Nb:STO substrate.

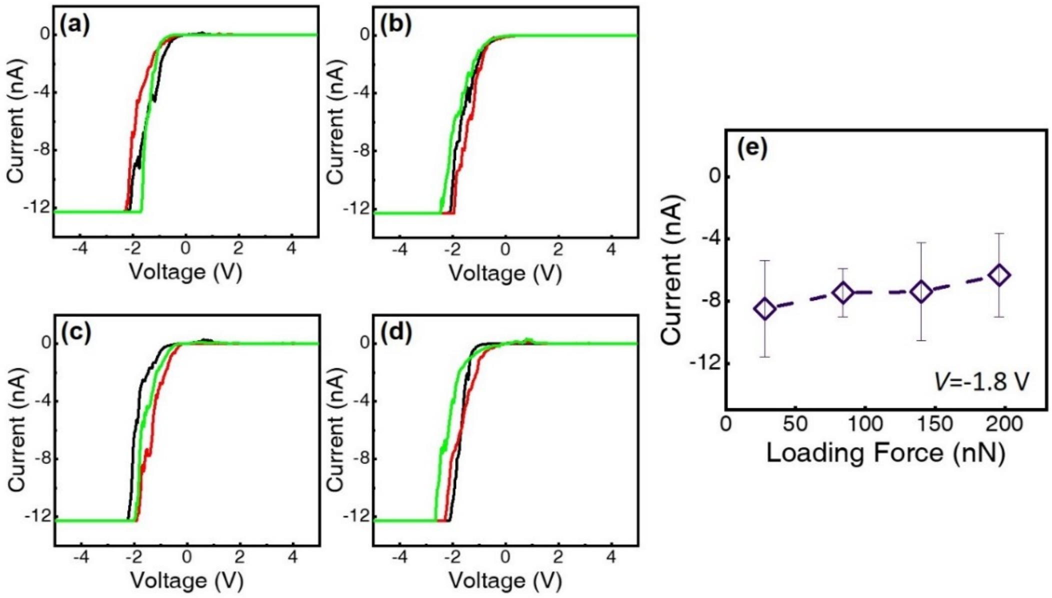

Figure S22 (a) to (d) *I-V* curve of the Pt/BTO junction with a tip-force of the stoichiometric BTO film under applied force of 28 nN, 84 nN, 140 nN and 196 nN, respectively. Red, yellow and black line refer to repeating measurements (three times) with the same conditions.

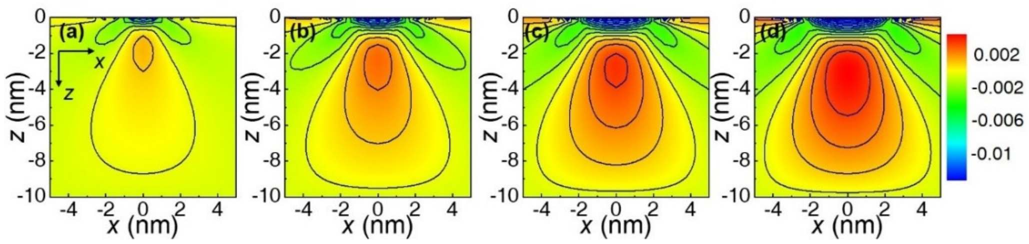

Figure S23 (a) to (d) COMSOL FEM calculation of the strain distribution with a tip-force model of the stoichiometric BTO film under applied force of 28 nN, 84 nN, 140 nN and 196 nN, respectively.

8. Strain distribution in 90 nm-thick film

In order to understand the effect of thickness on the flexoelectric effect of $BTO_{3-\delta}$, COMSOL FEM calculation was used to estimate the strain distribution in $BTO_{3-\delta}$ films with a thickness of 90 nm. As shown in Figure S24, when the same load force applied to the film, the strain occurs in a larger region in the 90 nm-thick film, and the maximal strain value is less than the strain value in the 10 nm-thick film.

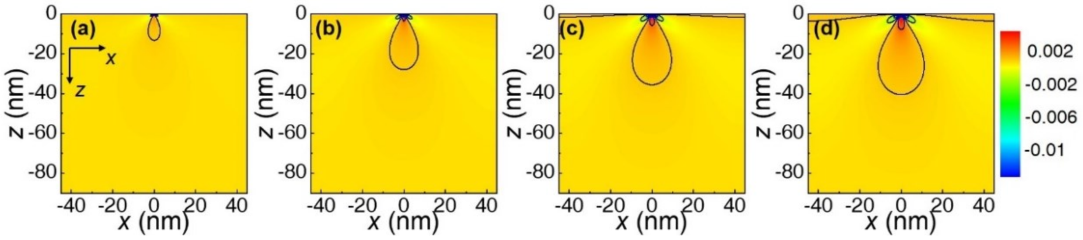

Figure S24 (a) to (d) COMSOL FEM calculation with a tip-force model of the 90 nm-thick $BTO_{3-\delta}$ film under applied force of 28 nN, 84 nN, 140 nN and 196 nN, respectively.

9. Simulation of the strain distribution and calculation of GF

The radius a and depth h of contact area under load *F* under Hertz model are given by:

$$a = \sqrt{Rh}$$

$$h = \left(\frac{3F}{4E\sqrt{R}}\right)^{2/3}$$

where *R* is tip radius 25 nm, *E* is effective Young's modulus. *E* is given by:

$$E = \frac{2E_{tip}E_{film}}{E_{film} * \left(1 - v_{tip}^2\right) + E_{tip} * \left(1 - v_{film}^2\right)}$$

where *v* is Poission's ratio.

The stress distribution can be simulated in a cylindrical coordinate, and the strain distribution can be calculated using Hooke's law:

$$\varepsilon_i = \frac{1}{E}[\sigma_i - \nu(\sigma_j - \sigma_k)]$$

$$\varepsilon_{ij} = \frac{1 + \nu}{E}\tau_{ij}$$

The parameter using in COMSOL simulation is:

Materials	Young's modulus	Poission's ratio	Reference
$BTO_{3-\delta}$	115 GPa	0.22	This work measured
BTO	170 GPa	0.23	[8]

Based on the simulation of the strain distribution, the maximum strain of each loading force can to calculated. So that the $\Delta\varepsilon$ can be calculated. Using the fomular GF=$(\Delta I/I)/\Delta\varepsilon$ can obtain the GF.

10. Comparison of flexoelectricity of various oxide materials

The flexoelectric properties of common oxide materials are list in Table S1. For flexoelectric property, both gauge factor (GF) and flexoelectric coefficient (with a unit of μC/m) are used in the literature.

Table S1 A comparison of flexoeletricity of various oxide materials.

No.	Materials	Thickness	Flexoelectric Property	Reference
1	$BaTiO_{3-\delta}$ thin film	~10 nm	1127 (GF)	This work
2	$BaTiO_3$ thin film	~10 nm	235 (GF)	This work
3	Nb:$SrTiO_3$ single crystal	0.5 mm	183 (GF)	[9]
4	$BaTiO_3$ single crystal	0.46 mm	2 μC/m	[10]
5	$BaTiO_3$ ceramic	-	10 μC/m	[11]
6	$BaTiO_3$ ceramic	5 mm	17.33 μC/m	[12]
7	$BaTiO_3$ ceramic @T_c	-	50 μC/m	[11]
8	$BaTiO_{3-\delta}$ single crystal	0.46 mm	1 mC/m	[10]
9	$SrTiO_3$ single crystal	0.3 mm	3 nC/m	[13]
10	$BiFeO_3$ thin film	~70 nm	1.4 μC/m	[14]

Reference

1. Li, P. *et al.* Direct growth of unidirectional graphene nanoribbons on vicinal Ge(001). *Phys. status solidi – Rapid Res. Lett.* **14**, 1900398 (2020).

2. Wang, T. *et al.* Wafer-scale fabrication of single-crystal graphene on Ge (110) substrate by optimized CH_4/H_2 ratio. *Appl. Surf. Sci.* **529**, 147066 (2020).

3. Coy-Diaz, H., Addou, R. & Batzill, M. Interface between graphene

and $SrTiO_3$ (001) investigated by scanning tunneling microscopy and photoemission. *J. Phys. Chem. C* **117**, 21006–21013 (2013).

4. Lee, C., Wei, X., Kysar, J. W. & Hone, J. Measurement of the elastic properties and intrinsic strength of monolayer graphene. *Science* **321**, 385–388 (2008).

5. Choi, K. J. *et al.* Enhancement of ferroelectricity in strained $BaTiO_3$ thin films. *Science* **306**, 1005–1009 (2004).

6. Niu, G. *et al.* Molecular beam epitaxy growth of $BaTiO_3$ thin films and crucial impact of oxygen content conditions on the electrical characteristics. *Thin Solid Films* **520**, 4595–4599 (2012).

7. Dai, L. *et al.* Toward van der Waals epitaxy of transferable ferroelectric barium titanate films via a graphene monolayer. *J. Mater. Chem. C* **8**, 3445–3451 (2020).

8. de Jong, M. *et al.* Charting the complete elastic properties of inorganic crystalline compounds. *Sci. Data* **2**, 150009 (2015).

9. Wang, L. *et al.* Flexoelectronics of centrosymmetric semiconductors. *Nat. Nanotechnol.* **15**, 661–667 (2020).

10. Narvaez, J., Vasquez-Sancho, F. & Catalan, G. Enhanced flexoelectric-like response in oxide semiconductors. *Nature* **538**, 219–221 (2016).

11. Ma, W. & Cross, L. E. Flexoelectricity of barium titanate. *Appl. Phys. Lett.* **88**, 2004–2007 (2006).

12. Hu, T., Deng, Q., Liang, X. & Shen, S. Measuring the flexoelectric coefficient of bulk barium titanate from a shock wave experiment. *J. Appl. Phys.* **122**, 055106 (2017).

13. Zubko, P., Catalan, G., Buckley, A., Welche, P. R. L. & Scott, J. F. Strain-gradient-induced polarization in $SrTiO_3$ single Crystals. *Phys. Rev. Lett.* **99**, 99–102 (2007).

14. Cheng, C. E. *et al.* Revealing the flexoelectricity in the mixed-phase regions of epitaxial $BiFeO_3$ thin films. *Sci. Rep.* **5**, 1–5 (2015).
